# Partnering with rural libraries to increase telehealth utilization in New York state

**DOI:** 10.5195/jmla.2025.2132

**Published:** 2025-10-23

**Authors:** Abdi T. Gudina, Sarah Merritt, Rohan Dhawan, Milena E. Insalaco, Joanne Kochanek, Mitzi Sackett, Stacey Wicksall, Lynae Wyckoff, Diana Huussen, Francisco Cartujano-Barrera, Ana Paula Cupertino, Michele Foster, Jason Coleman, Charles S. Kamen

**Affiliations:** 1 abdi_gudina@urmc.rochester.edu, University of Rochester, Rochester, NY; 2 sarah_merritt@urmc.rochester.edu, University of Rochester, Rochester, NY; 3 rdhawan@u.rochester.edu, University of Rochester, Rochester, NY; 4 University of Rochester, Rochester, NY; 5 jfk@rochester.rr.com, University of Rochester, Rochester, NY; 6 mitzi.sackett@bassett.org, Bassett Healthcare, NY; 7 swicksall@owwl.org, Macedon Public Library, Macedon, NY; 8 lwyckoff@nycamh.org, Bassett Healthcare, NY; 9 diana_huussen@urmc.rochester.edu, University of Rochester, Rochester, NY; 10 francisco_cartujano@urmc.rochester.edu, University of Rochester, Rochester, NY; 11 paula_cupertino@urmc.rochester.edu, University of Rochester, Rochester, NY; 12 michele.foster@s2aynetwork.org, Pivital Public Health Partnership, New York, NY; 13 jason.coleman@cancer.org, American Cancer Society, New York, NY; 14 charles_kamen@urmc.rochester.edu, Associate Professor, University of Rochester, Rochester, NY

**Keywords:** Telehealth, Community Engagement, Libraries, Rural, Disparities

## Abstract

**Background:**

New York State (NYS) residents living in rural communities experience multiple barriers to accessing healthcare. Telehealth, or remote provision of healthcare services, could address these barriers. However, telehealth remains underutilized in rural communities due to limited access to broadband and lack of provider/patient awareness. Rural libraries could serve as telehealth hubs and thereby increase telehealth uptake.

**Case Presentation:**

A community-academic partnership was formed between the University of Rochester Wilmot Cancer Institute and the Community Cancer Action Council, a group of 29 community stakeholders. The partnership surveyed libraries across NYS to assess telehealth capacity. After identifying a library to pilot a telehealth hub, surveys were sent to that library's patrons and staff to assess perspectives on telehealth. Fifty-three libraries (19.4%) responded to the initial survey, 92.2% of whom felt libraries could beneficially host telehealth hubs. The Macedon Public Library was chosen as the pilot location as they had constructed a private telehealth booth. 60% of 48 Macedon community members surveyed indicated they would utilize telehealth in the library, while 89% of 9 Macedon library staff agreed they were committed to implementing telehealth services.

**Conclusions:**

We found high community interest in establishing a community telehealth hub in a library. In the next phase of the project, the community-academic partnership will promote use of telehealth to oncology providers.

## BACKGROUND

Geographic access to healthcare is a barrier experienced by many New York State residents, as 86.6% of New York State land is considered rural [1]. Individuals living in rural communities may need to travel long distances to access specialty healthcare services, and may incur additional financial and practical costs in doing so [1]. Telemedicine is a potential solution to overcome these barriers. The World Health Organization defines telemedicine as the delivery of healthcare services using technological means to overcome access barriers related to distance, with the goal of diagnosing, treating, preventing, educating about, and researching disease and injuries [2]. Telehealth appointments, a form of telemedicine, became especially popular during the COVID-19 pandemic in 2020. With mandatory isolation, telehealth healthcare visits became an essential way to deliver care without the risk of spreading COVID-19, and were reimbursed at the same level as in-person visits [3, 4].

Even as mandatory isolation policies eased, many healthcare systems have found that telehealth has the potential to improve access to healthcare services, reduce healthcare costs, and increase patient satisfaction [5–8]. Using telehealth, providers can deliver remote healthcare services, such as virtual consultations and health literacy programs, to geographically remote rural communities [9]. In this manuscript, we use the US Census Bureau definition of “rural,” meaning areas that are neither urbanized (with more than 50,000 residents) nor urban clusters (with between 50,000 and 2,500 residents). Despite its benefits, the implementation of telehealth in rural areas faces several challenges, including limited access to broadband infrastructure and technological resources, lack of knowledge and acceptance of telehealth services [10–13]. To fully harness the potential of telehealth in rural settings, it will be necessary to address these challenges through the development of appropriate technological infrastructure, increasing awareness and acceptance of telehealth services among healthcare providers and patients, and developing sustainable reimbursement models [14, 15].

Prior research has examined potential solutions to the issue of telehealth infrastructure in rural areas [16, 17]. Libraries, often considered trusted community centers, can be ideal locations to deploy telehealth services [18]. By establishing such community sites, rural residents can access reliable high-speed internet connectivity and technological resources necessary for telehealth services like private telehealth booths, computers, webcams, and even telehealth-related software. Libraries can also play an essential role in promoting awareness, accessibility, and acceptance of telehealth services by providing education, training, and advertising to patients and providers [19, 20]. Given that many vulnerable communities find libraries to be accessible and trustworthy, researchers and community partners have called on practitioners and policy makers to implement more public health services in libraries [21–24]. Librarians and library partners have already implemented a variety of programs to increase access to their communities for a variety of direct healthcare services, health resources, and linkage to services [22, 24, 25]. Some of these programs include supplying Narcan to manage opioid overdose in their community or staffing a COVID-19 help hotline [24, 25]. With many libraries already offering other healthcare services, this positions them well to effectively provide telehealth services. One recent study queried whether public libraries could be a potential access point for telehealth visits [19]. Fifteen librarians from nine states participated in semi-structured interviews that asked about the communities where the libraries were located, the types of patrons typically frequenting the libraries, and the librarians' views on barriers and benefits of public libraries as access locations for telehealth. This study indicated that librarians support libraries as telehealth access locations, that libraries face financial barriers to telehealth implementation, and that rural public health nurses can be a key component in collaborative efforts between regional libraries and healthcare networks. These results indicate the need for further research focused specifically on telehealth implementation in rural public libraries.

## CASE PRESENTATION

We sought to understand the perspectives of rural library staff and rural community members on implementing a telehealth hub in a library. To that end, we conducted a survey of staff at public libraries across the University of Rochester Wilmot Cancer Institute (WCI)'s catchment area to assess the knowledge, interest, and capacity of these libraries to provide access to telehealth services. We then chose a rural library in Macedon, NY from among the survey respondents to serve as a pilot location for providing telehealth access. We surveyed library staff and patrons about their perspectives on telehealth and what next steps would be needed to implement a telehealth hub at the selected library.

### Study Design and Procedure

This study was led by a community-academic partnership between the University of Rochester WCI and the Community Cancer Action Council (CCAC), a group of 29 community stakeholders. The partnership identified the need for increased access to healthcare in rural areas within WCI's catchment area. The WCI's catchment area consists of a 27-county area of Western and Central New York that includes more than 3 million people in the Finger Lakes, Southern Tier, Central, and Mohawk Valley regions. The study aimed to provide a holistic understanding of the possibility of providing telehealth access within a public library. A series of surveys were developed and disseminated in four phases. Phases were completed between August 2021 and December 2022. There were no incentives for completing any of the surveys across phases. The University of Rochester Research Subjects Review Board approved all phases.

#### **Phase 1:** NYS libraries & telehealth survey (library survey)

The community-academic team developed the survey and circulated it to libraries within the WCI's catchment area to identify telehealth capacity and determine a pilot location for this project. The survey was sent to the libraries through email addresses available on the New York State Public Library System email directory listserv, and direct outreach via email addresses available on the library website. All libraries within the WCI's catchment area received an email from the study team explaining the purpose of the study and including a link to the REDCap survey. These emails were sent directly from the REDCap system. A total of 273 libraries were invited to participate in the survey, with 53 completing the survey (a 19.4% response rate).

#### **Phase 2:** Community patron survey (community survey):

**Figure 1:**
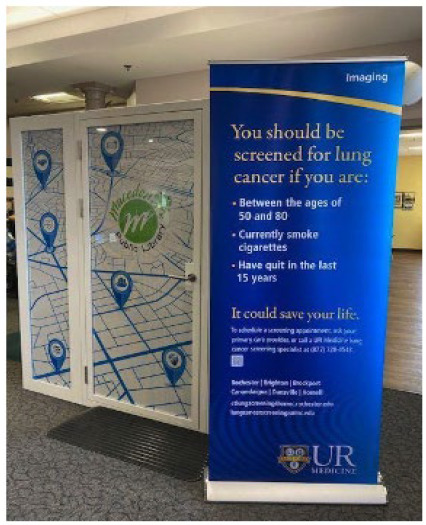
Digital equity booth at the Macedon Public Library, including materials from the community-academic partnership with University of Rochester.

Three respondents to the library survey indicated that they were in the process of implementing a telehealth hub using grant funding from New York State. A member of the study team contacted the director of each of these libraries to determine whether any would be willing to conduct additional surveys to assess their telehealth implementation process. The director of the Macedon Public Library expressed interest in participating as a pilot site for telehealth hub implementation. Macedon is a rural town in Wayne County, New York, with a population of approximately 9,000 people. The Macedon Public Library had recently constructed a “digital equity booth” with grant funding from New York State (see Figure 1 and Supplemental Materials). This booth was located in the foyer of the library; it included a computer, headset, and camera; it had space for two people to sit comfortably; and it was soundproof and opaque from the outside, ensuring privacy. In partnership with the library director, the study team developed a community survey to assess Macedon community members' and Macedon Public Library patrons' perspectives on telehealth and the use of the digital equity booth to enhance access to telehealth.

To disseminate the community survey, the study team attended two community events in collaboration with the Macedon Public Library: the *Macedon Heritage Festival* and the *Macedon Senior Luncheon*. Participants at each event were residents of Macedon and the surrounding area who are either existing or potential Macedon Public Library patrons. Participants completed a paper version of the survey, which the study team entered into REDCap. A total of 48 community participants completed the survey.

#### **Phase 3:** Library staff survey (library staff survey)

The community-academic team developed the third survey using the ORIC Readiness for Implementing Change (ORIC) scale [26]. This survey targeted Macedon Public Library staff to assess their readiness to implement a telehealth hub. Participants were recruited at an employee meeting by the study team. Participants completed a paper version of the survey, and the survey team entered the data into REDCap. All three full-time library staff and six of the nine part-time library staff completed the survey. All responding staff were responsible for scheduling visits at the hub, answering patron questions about the hub, and participating in promotion of the hub, and so were able to respond to the ORIC questions.

#### **Phase 4:** Vendor feedback survey (vendor survey)

The study team organized a health fair at the Macedon library to improve awareness of the telehealth hub. The study team invited local community organizations that utilize telehealth or digital health services within their work to join the event and display their services. The study team also developed a vendor feedback survey to assess the event's effectiveness at promoting telehealth at the library. The vendor survey included three closed-ended, multiple choice (YES or NO) questions. Thirteen community organization vendors attended the event, and seven completed the survey.

### Participants

As surveys were anonymous and multi-level, sociodemographic characteristics of each participant were not collected for this study. Participants eligible to participate in this study were required to be at least 18 years old, possess English language proficiency, and either live, work or volunteer within the WCI's catchment area. Specifically, the library survey required participants to volunteer or be employed at a NYS library within the WCI's catchment area. The library staff survey required participants to volunteer or be employed at Macedon Public Library. The vendor survey required attendance at the Macedon Public Library health fair event.

Participants for all surveys were given an information sheet outlining research objectives, study procedures and contact information for both the principal investigator and the study coordinator if questions arose. Those willing to participate in the library and vendor surveys selected “Accept” at the conclusion of the information sheet on REDCap, before being redirected to complete the survey. Those willing to participate in the community and library staff surveys completed paper versions of these surveys after receiving the information sheet.

### Capability and infrastructure of libraries to host telehealth *(Phase 1*)

From the library survey, data on the utilization of telehealth sites, existing infrastructure, and possibilities of community engagement with telehealth services was compiled in **Table 1**. Notably, while the vast majority of responding libraries reported that a telehealth site in a community setting would be beneficial and that they would be willing to promote health initiatives and digital health services through the library, a much smaller percentage reported that they would be interested in leading the implementation of a telehealth site in their organization, and a small minority of respondents stated that they had an established partnership with a local health system or doctors (see **Table 1**).

**Table 1:** Assessing capability and infrastructure of libraries to host telehealth

Do you think a telehealth site in a community location would be beneficial?	Total n=53 (100%)
Yes	49 (92.5)
No	4 (7.5)
Would you personally feel comfortable using a telehealth site in a community location?	Total n=53 (100%)
Yes	39 (73.6)
No	14 (26.4)
Would people in your community use a telehealth site in a community location?	Total n=51 (100%)
Yes	45 (88.2)
No	6 (11.8)
Would your organization be interested in hosting a telehealth site?	Total n=53 (100%)
Yes	43 (81.1)
No	10 (18.9)
Do you have a private space in your facility that could be used to host a community telehealth site?	Total n=43 (100%)
Yes	34 (79.1)
No	9 (20.9)
Is there anyone who would be interested in being a point person/lead/champion for setting up a telehealth site in your organization?	Total n=41 (100%)
Yes	29 (70.7)
No	12 (29.3)
Do you feel there would be participation among local doctors to provide services through a telehealth site in a community location?	Total n=43 (100%)
Yes	36 (88.7)
No	7 (16.3)
Do you have a current working relationship with a local health system or local doctors?	Total n=51 (100%)
Yes	9 (17.6)
No	42 (82.4)
Is there a place at your facility where a training for community members about using telehealth could be held (e.g., classroom, conference room)?	Total n=54 (100%)
Yes	47 (87.0)
No	7 (13.0)
Would you be willing to promote other health initiatives and digital health services if they were offered (cancer screenings, wellness workshops, cooking demonstrations, etc.)?	Total n=52 (100%)
Yes	50 (96.2)
No	2 (3.8)

* As the number of respondents vary by survey items, the denominator used for percentage calculation also varies by survey item (range: 41-54)

### Community members' perspectives on telehealth utilization at the Macedon Public Library (*Phase 2*)

From the community survey, community members' prior experiences with telehealth, comfort levels in utilizing the booth, potential concerns and overall satisfaction with the booth were measured by a Likert scale and compiled in **Table 2**. Existing experience with telehealth was limited, with the minority of respondents stating that they had a telehealth appointment via computer or phone, and nearly half (45.8%) stating that they never used telehealth before. However, the majority of patients stated that they would feel comfortable going to the Macedon Public Library for a telehealth appointment and that they had no concerns about using a telehealth booth in the Macedon library (see **Table 2**).

**Table 2:** Assessing patrons' perspectives on telehealth utilization at the Macedon Library (n*)

Have you ever had a telehealth appointment?	Total n = 48 (100%)
Yes, on the computer	13 (27.1)
Yes, on the phone	13 (27.1)
No	22 (45.8)
Would you feel comfortable going to a telehealth appointment at the Macedon library?	Total n = 47 (100%)
Yes	29 (61.7)
No	18 (38.3)
Do you have concerns about using a telehealth booth in the Macedon library?	Total n = 45 (100%)
Yes	8 (17.8)
No	37 (82.2)
How satisfied are you with the telehealth booth at the Macedon library?	Total n = 19 (100%)
Very Dissatisfied	0 (0.0)
Somewhat Dissatisfied	0 (0.0)
Neither Satisfied nor Dissatisfied	5 (26.3)
Somewhat Satisfied	1 (5.3)
Very Satisfied	13 (68.4)

### Readiness to implement a telehealth booth *(Phase 3)*

Macedon Public Library staff (n=9) were given the *library staff survey* during their routine shifts to assess their readiness to implement a telehealth booth at the library (see **Table 3**). In total, 55.5% agreed or somewhat agreed that the organization could get people invested in implementing a telehealth booth; 88.9% agreed or somewhat agreed that they want to implement telehealth booth; 88.9% agreed or somewhat agreed that they were committed to implementing a telehealth booth; and 66.6% agreed or somewhat agreed that they could handle the challenges that might arise in implementing a telehealth booth. Moreover, the vast majority (88.9%) of the participants agreed or somewhat agreed that they could coordinate tasks so that implementation goes smoothly and 77.7% agreed or somewhat agreed that they were motivated to implement a telehealth booth.

**Table 3:** Organizational Readiness for Implementing a Telehealth booth at Macedon library (n=9)

	Disagree, N = 9 (100%)	Somewhat Disagree, N = 9 (100%)	Neither Agree nor Disagree, N = 9 (100%)	Somewhat Agree, n N = 9 (100%)	Agree, N = 9 (100%)
I feel confident that the organization can get people invested in implementing a telehealth booth.	0 (0.0)	0 (0.0)	4 (44.4)	3 (33.3)	2 (22.2)
**I am committed to implementing a telehealth booth.**	**0 (0.0)**	**0 (0.0)**	**1 (11.1)**	**5 (55.6)**	**3 (33.3)**
I feel confident that we can keep track of progress in implementing a telehealth booth.	0 (0.0)	0 (0.0)	1 (11.1)	5 (55.6)	3 (33.3)
I will do whatever it takes to implement a telehealth booth.	0 (0.0)	0 (0.0)	2 (22.2)	3 (33.3)	4 (44.4)
I feel confident that the organization can support people as they adjust to using a telehealth booth.	0 (0.0)	0 (0.0)	2 (22.2)	4 (44.4)	3 (33.3)
**I want to implement a telehealth booth.**	**0 (0.0)**	**0 (0.0)**	**1 (11.1)**	**6 (66.7)**	**2 (22.2)**
I feel confident that we can keep the momentum going in implementing a telehealth booth.	0 (0.0)	0 (0.0)	0 (0.0)	5 (55.6)	4 (44.4)
**I feel confident that we can handle the challenges that might arise in implementing a telehealth booth.**	**0 (0.0)**	**0 (0.0)**	**3 (33.3)**	**2 (22.2)**	**4 (44.4)**
I am determined to implement a telehealth booth.	0 (0.0)	0 (0.0)	4 (44.4)	4 (44.4)	1 (11.1)
**I feel confident that we can coordinate tasks so that implementation goes smoothly.**	**0 (0.0)**	**0 (0.0)**	**1 (11.1)**	**5 (55.6)**	**3 (33.3)**
**I am motivated to implement a telehealth booth.**	**0 (0.0)**	**0 (0.0)**	**2 (22.2)**	**4 (44.4)**	**3 (33.3)**
I feel confident that we can manage the politics of implementing a telehealth booth.	0 (0.0)	0 (0.0)	5 (55.6)	2 (22.2)	2 (22.2)

### Library as a venue for promoting telehealth (*Phase 4*)

Of the 13 vendors at the Macedon Public Library health fair, 7 completed the vendor survey. In total, 71% of vendors reported that the fair was successful in promoting telehealth, 43% received referrals to their programs or services, and 100% of vendors would attend another community event at the Macedon Public Library, signifying the event was constructive and beneficial.

## DISCUSSION

Our study assessed the capability of libraries in the WCI's catchment area and the perspectives and readiness of library staff and the local community to implement telehealth at a pilot library, Macedon Public Library. Among the libraries that participated in the survey, the majority of them reported that they have the capacity and infrastructure to host telehealth within their respective libraries. Local community residents (who are potential users of the telehealth) surrounding the Macedon Library reported that they support the idea of implementing telehealth within the library. Moreover, the staff at Macedon Library (who oversee the library) expressed their readiness to implement a telehealth booth within Macedon Public Library.

Infrastructure is a critical component of implementing telehealth hubs in rural communities. Assessing the availability and robustness of community infrastructure is crucial for the successful implementation and delivery of telehealth services, as the lack of infrastructure is a global challenge that healthcare organizations are facing as they attempt to integrate telehealth into their workflows [27]. Most libraries in the WCI's catchment area, however, have critical components of telehealth infrastructure that could enable them to implement telehealth hubs within their libraries. Specifically, in addition to broadband internet, availability of private space and presence of a staff champion are essential components of infrastructure for telehealth [28]. The pilot library chosen, Macedon Public Library, reported these important infrastructure features, including a private telehealth booth and a conference room where training for library staff and community members about utilization of telehealth could be held. In addition, two other libraries in the WCI's catchment area reported the capacity to construct a private booth to serve as a telehealth hub, indicating that this infrastructure exists across multiple libraries. Healthcare organizations could leverage the immense potential of public libraries to extend their service delivery coverage to a wider range of communities, particularly to rural communities who may experience limited access to healthcare facilities, barriers to transportation, and lack of broadband internet at their homes.

Various stakeholders are involved in telehealth implementation and utilization including patients, medical providers, insurance payors, and policymakers [29]. Although each of these stakeholders plays their respective role, the perspectives of the local communities (i.e., the ultimate users of the services) are the prime determinant of telehealth uptake. Hence, assessing the perspectives of the local community regarding implementing telehealth hubs is crucial before starting the services. Among the local community participants surrounding the Macedon Public Library who participated in the survey, close to three-quarters of them reported their interest in using telehealth at Macedon Library. They also reported that they felt comfortable having a telehealth appointment at Macedon library. Finally, successful implementation of telehealth hubs within public libraries requires sustained engagement of library staff. The library staff members at the Macedon Public Library expressed a strong commitment and readiness to implement telehealth hub within the library. Again, this indicates that healthcare organizations could leverage the readiness of these communities and staff members when strategizing around telehealth implementation.

Integrating telehealth into public libraries requires cooperation between multiple stakeholders, including health systems, community-based organizations, and libraries, to provide culturally tailored and holistic care [20, 28]. It is notable from our results that while 87% of libraries reported that they had space to host a telehealth hub, 82% did not have an existing relationship with healthcare organizations or providers. Developing these relationships may further enhance telehealth referral pipelines into community-based hubs. Offering targeted telehealth training within libraries, using locally-familiar doctors, and marketing the presence of library-based telehealth hubs to both doctors and patients could bolster utilization of these hubs. Other ways to engage diverse stakeholders in implementing telehealth could include focusing on particular segments of the population (ex: economically disadvantaged, elderly, chronically ill) to ensure they are aware of and able to access community telehealth hubs, and training patients and community members on the use of online healthcare accounts (e.g., patient portals). As a community-academic partnership hosted by a cancer center, the CCAC was particularly interested in the potential for rural telehealth hub to promote access to cancer care. Promoting telehealth access to cancer care would involve developing specific relationships with oncology providers and specific education for the community about use of telehealth across the continuum of cancer care services.

Our study, while providing insight into the feasibility and perspectives of rural libraries as telehealth hubs, is not without limitations. The response rate from the initial statewide library survey was low, with only 51 out of 274 libraries responding. Though this matches rates in other online surveys, it potentially introduces a selection bias where libraries with either a pre-existing inclination or familiarity towards telehealth might have been overrepresented. The use of self-reported data raises the risk of either recall bias or social desirability biases, especially when highlighting the potential benefits of telehealth. Further, individual respondents may have been more interested in the idea of telehealth than those who opted not to respond. Though our community survey included a definition of telehealth, individuals with low health literacy may not have fully understood the uses of telehealth. The sample from the Macedon Public Library, which included both patrons and staff, was relatively small, potentially not capturing all of the perspectives in the larger community and limiting the generalizability of the results. To expand on this point, it is unclear whether the results from a single pilot location at the Macedon Library can be applied to other rural libraries, both within and outside New York State. This study did not thoroughly assess the telehealth hub's long-term feasibility and sustainability. Additionally, for certain measures, we encountered limited responses, and notably, the vendor feedback from the health far did not yield many substantive insights for our analysis. We did not collect demographics from respondents to preserve their anonymity, thus further limiting our ability to assess generalizability of results. In order to ensure a long-lasting and successful integration of telehealth hub's in rural libraries, future initiatives should aim for more comprehensive study of these factors. Despite these limitations, our sampling of libraries was robust, our response rate was acceptable, and our community-academic partnership proved both feasible and effective in conducting the project.

In conclusion, public libraries have the capacity (i.e., private rooms and other important infrastructure factors) to serve as telehealth sites. Moreover, the local community and the staff members at our pilot public library, Macedon Public Library, support telehealth implementation within the library. Healthcare organizations should harness such great resources of public libraries (particularly in rural areas) to address disparities in healthcare service coverage.

## Data Availability

All data from surveys conducted as part of this article are available in the Open Science Framework at DOI 10.17605/OSF.IO/TD3GA.
