# Supplementary material for: Partnering with rural libraries to increase telehealth utilization in New York state
Source: J Med Libr Assoc. 2025 Oct 23;113(4):349–57. doi: 10.5195/jmla.2025.2132 (PMC12604070; doi:10.5195/jmla.2025.2132)
Supplement: Supplementary file 1 — Appendix A [file jmla-113-4-349-s01.pdf]

Community Telehealth Site Survey

INFORMATION SHEET

Community-Cancer-Connect: A Survey About Telehealth in Rural Communities

Principal Investigator: Charles Kamen, PhD

This form describes a research study that is being conducted by Charles Kamen, PhD from the University of Rochester's Department of Surgery.

The purpose of this study is to better understand the perspectives of community organizations about providing telehealth to the communities that they serve.

If you decide to take part in this study, you will be asked to complete a survey.

We estimate that approximately 82 volunteers will take part in this study. Your participation will last about 5-10 minutes.

There are no other expected risks to you for participating in this study. There are also no expected benefits. You will not be paid for participating in this study.

The University of Rochester makes every effort to keep the information collected from you private. In order to do so, we will make sure only approved research staff members have access to study information collected. If you choose to provide contact information, such as your organization's phone number or address, your survey responses may no longer be anonymous to the researcher. However, no names or identifying information will be included in any publications or presentations based on these data, and your responses to this survey will remain confidential.

Your permission to use your survey data for this study will not expire unless you tell us you want to cancel it. We will keep the information we collect about you indefinitely. If you cancel your permission, you will be removed from the study.

Your participation in this study is completely voluntary. You are free not to participate or to withdraw at any time, for whatever reason. No matter what decision you make, there will be no penalty or loss of benefits to which you are otherwise entitled.

For more information or questions about this research you may call Sarah Merritt
at (585) 738-5539. Please contact the University of Rochester Research Subjects Review Board at 265 Crittenden Blvd., CU 420628, Rochester, NY 14642, Telephone (585) 276-0005 or (877) 449-4441 for the following reasons:
• You wish to talk to someone other than research staff about your rights as a research subject;
• To voice concerns about the research;
• To provide input concerning the research process;
• In the event the study staff could not be reached.

Do you agree to participate in this study?

○ Yes
○ No

We are interested in your thoughts about setting up a telehealth site in a community location. A telehealth site would provide patients access to a private space with a computer, webcam, microphone, a stable internet connection, and other equipment to allow them to connect via the internet to a healthcare provider. A community location could be a library, senior living center, school, or other public space that could host this telehealth site.

Do you think a telehealth site in a community location would be beneficial?
- ◯ Yes
- ◯ No

Choose top three locations in your community that could best host a telehealth site (select multiple):
- ☐ library
- ☐ senior center
- ☐ community center
- ☐ school
- ☐ recreation center
- ☐ church
- ☐ firehall
- ☐ Office for Aging
- ☐ other

Fill in other locations for a telehealth site: ______________________________

Would you personally feel comfortable using a telehealth site in a community location?
- ◯ Yes
- ◯ No

Why not? ______________________________

Would people in your community use a telehealth site in a community location?
- ◯ Yes
- ◯ No

Why not? ______________________________

Would your organization be interested in hosting a telehealth site?
- ◯ Yes
- ◯ No

Do you have a private space in your facility that could be used to host a community telehealth site?
- ◯ Yes
- ◯ No

Is there anyone who would be interested in being a point person/lead/champion for setting up a telehealth site in your organization?
- ◯ Yes
- ◯ No

Name: ______________________________

Email address: ______________________________

Is there anyone within your organization who would need to approve hosting a telehealth site at your location? Names and titles: ______________________________

What resources do you already have that could make a telehealth site work at your organization?

- ☐ designated room
- ☐ furniture
- ☐ computer
- ☐ good camera and lighting
- ☐ headphones
- ☐ infection prevention supplies
- ☐ scheduling assistant/software
- ☐ white noise machine
- ☐ scale
- ☐ blood pressure cuff
- ☐ training for staff
- ☐ educational resources for the community
- ☐ library champion
- ☐ other

What other resources do you have?

____

What would be your concerns about offering telehealth in a community setting? (select multiple):

- ☐ privacy
- ☐ transportation
- ☐ scheduling
- ☐ prefer in person
- ☐ lack of familiarity with technology/computer knowledge
- ☐ language barrier
- ☐ handicapped accessibility
- ☐ hours
- ☐ COVID or other illness
- ☐ other

Fill in other concerns:

____

Do you feel there would be participation among local doctors to provide services through a telehealth site in a community location?

- ◯ Yes
- ◯ No

Do you have a current working relationship with a local health system or local doctors?

- ◯ Yes
- ◯ No

Please describe this relationship:

____

What types of services would you want to see offered through this telehealth site?

- ☐ primary care
- ☐ mental health
- ☐ endocrinology
- ☐ urology
- ☐ oncology
- ☐ tobacco and substance use services
- ☐ integrative care (mindfulness, guided imagery)
- ☐ genetic counseling
- ☐ other

What other types of services would you want to see offered through a telehealth site?

____

Is it difficult (because of distance, availability, etc.) for people in your community to receive:

- ☐ Well visits or check-ups
- ☐ Sick visits or urgent care
- ☐ Mental health services
- ☐ Cancer prevention
- ☐ Specialty follow-up
- ☐ Other

What other services are difficult for your community to receive?

____

Please estimate: What percent of the community in which you live does not have reliable access to the internet?

0% 50% 100%

(Place a mark on the scale above)

Is there a place at your facility where a training for community members about using telehealth could be held (e.g., classroom, conference room)?

- ○ Yes
- ○ No

Would you be willing to promote other health initiatives and digital health services if they were offered (cancer screenings, wellness workshops, cooking demonstrations, etc.)?

- ○ Yes
- ○ No

Who in your community might not be able to access this telehealth site (e.g., those who are disabled, too sick, non-English speakers)?

____

What are other advantages of having a telehealth site in a community location?

____

What are other disadvantages of having a telehealth site in a community location?

____

What other comments do you have about setting up a telehealth site in a community location?

____

Your name:

____

Email address:

____

Organization:

____

Your organization's zip code:

____

Library Staff Survey

Please complete the survey below.Thank you!

Organizational Readiness for Implementing Change for Telehealth

		Disagree	Somewhat Disagree	Neither Agree nor Disagree	Somewhat Agree	Agree
1)	I feel confident that the organization can get people invested in implementing a telehealth booth.	○	○	○	○	○
2)	I am committed to implementing a telehealth booth.	○	○	○	○	○
3)	I feel confident that we can keep track of progress in implementing a telehealth booth.	○	○	○	○	○
4)	I will do whatever it takes to implement a telehealth booth.	○	○	○	○	○
5)	I feel confident that the organization can support people as they adjust to using a telehealth booth.	○	○	○	○	○
6)	I want to implement a telehealth booth.	○	○	○	○	○
7)	I feel confident that we can keep the momentum going in implementing a telehealth booth.	○	○	○	○	○
8)	I feel confident that we can handle the challenges that might arise in implementing a telehealth booth.	○	○	○	○	○
9)	I am determined to implement a telehealth booth.	○	○	○	○	○
10)	I feel confident that we can coordinate tasks so that implementation goes smoothly.	○	○	○	○	○
11)	I am motivated to implement a telehealth booth.	○	○	○	○	○
12)	I feel confident that we can manage the politics of implementing a telehealth booth.	○	○	○	○	○

Macedon Library Patron Survey

Please complete the survey below.Thank you!

People sometimes find it hard to get to the doctor's office. A telehealth appointment is an easy way to talk with your health care team by computer or phone. To provide you with access to these services, the Macedon Library has a private room, known as a telehealth booth, that can be used for telehealth appointments.

Have you ever had a telehealth appointment?
- ◯ Yes, on the computer
- ◯ Yes, on the phone
- ◯ No

Would you feel comfortable going to a telehealth appointment at the Macedon library?
- ◯ Yes
- ◯ No

Why not? ______________________________

Do you have concerns about using a telehealth booth in the Macedon library?
- ◯ No
- ◯ Yes

What concerns do you have? ______________________________

In your opinion, who would benefit the most in your community from using this telehealth booth? ______________________________

What are advantages of having a telehealth booth in the Macedon library? ______________________________

What are disadvantages of having a telehealth booth in the Macedon library? ______________________________

Do you have any other comments about setting up a telehealth booth in the Macedon library? ______________________________

	Very Dissatisfied	Somewhat Dissatisfied	Neither Satisfied nor Dissatisfied	Somewhat Satisfied	Very Satisfied
How satisfied are you with the telehealth booth at the Macedon library?	◯	◯	◯	◯	◯

ThanksLiving Vendor Feedback

Please complete the survey below.Thank you!

Organization Name: __________________________________

Contact Name: __________________________________
(Your name, or best person to contact at your organization)

Contact Email: __________________________________
(Your email, or best email for contacting your organization)

Overall, what did you think of the Thanksliving event at the Macedon Public Library, on November 17, 2022? ____

Did you get any referrals for your organization?
- ◯ Yes
- ◯ No

If so, how many? __________________________________

Do you think telemedicine was promoted well at this event?
- ◯ Yes
- ◯ No

Why not? ____

Would you attend another community event as a vendor at the Macedon Public Library?
- ◯ Yes
- ◯ No

Do you have any ideas to help promote the use of the digital privacy booth at the Macedon Public Library? ____
