# Supplementary material for: Partnering with rural libraries to increase telehealth utilization in New York state
Source: J Med Libr Assoc. 2025 Oct 23;113(4):349–57. doi: 10.5195/jmla.2025.2132 (PMC12604070; doi:10.5195/jmla.2025.2132)
Supplement: Supplementary file 2 — Appendix B [file jmla-113-4-349-s02.pdf]

30 Main Street
Macedon, NY 14502

RRLC DIGITAL EQUITY BOOTH REPORT

STATISTICS:

We have had **78 people** use the digital privacy booth since the end of June 2022.

The booth has been used for a variety of purposes. When patrons or staff come to use the booth, they are asked which category best describes their purpose:

- Health ***9 people***
- Legal ***0 people***
- Employment ***0 people***
- Social Services ***7 people***
- Support ***37 people All people in this category received help from Literacy Rochester Digital Literacy navigators.***
- Educational ***8 people***
- Zoom or other meeting ***10 people***
- Financial ***0 people***
- Would rather not comment* ***7 people***

We had some people who preferred not to categorize their use and we respected their wishes as per our policy.

POLICY/PROCEDURE

Here is the board approved policy created to manage the telehealth booth:

—---

Section D: Operations **D-24**
Subsection: Digital Privacy Booth Policy

The Macedon Public Library offers a digital privacy booth to patrons. The booth is offered to make private, digital meetings accessible to all.

The booth can offer complete privacy via the use of a computer with a camera and headset if a patron types using the keyboard to communicate via online chat. If a patron opts to speak aloud

using the headset microphone, the booth is semi-private (the sound is muffled, but not completely blocked). Patrons may choose their level of privacy at their own personal discretion.

The uses of the booth may include instances such as:

- Health navigator appointments
- Telehealth appointments
- Virtual job interviews
- Social services appointments
- Therapeutic appointments
- Webinars and virtual training opportunities
- Zoom meetings to stay connected to family
- Virtual classes
- Online support group meetings such as Alcoholics Anonymous or Weight Watchers
- Legal consultations
- Tax preparation appointments

The booth may be booked in one hour blocks of time with a limit of no more than three hours of time blocked per patron in any one day. Appointments must be booked a minimum of one day prior to the appointment by calling the library.

The library reserves the right to schedule blocks of time for special outreach services such as health navigator appointments or free tax preparation services.

All appointments must be coordinated by the patron. If a patron has further questions following the appointment, the patron will be responsible for reaching out to the person or people they engaged with virtually.

The library assumes no responsibility or liability for the quality of the appointment the patron receives as this is a meeting that is between the patron and a third party.

The library will provide the technology, equipment and internet connectivity necessary for a virtual appointment to occur, but assumes no liability for failure of the technology, equipment or internet connectivity on the date of the appointment.

The library will maintain the confidentiality of all patrons who opt to use the booth. No record of any individual appointment shall be kept on file.

The library will count each appointment type in aggregate for the purpose of providing statistical information for state reporting only. No individual name shall be attached to any reporting of statistics.

The categories for reporting appointments are:

- Health
- Legal

- Employment
- Social services
- Support
- Educational
- Financial
- Personal

Patrons will be asked to categorize their appointment under one of these broad categories to further protect privacy. ***No patron will be asked to give any descriptive details associated with their appointment.***

—--

<u>APPOINTMENTS:</u>

We kept track of appointments and their purpose using the following paper form kept on a clipboard at the circulation desk:

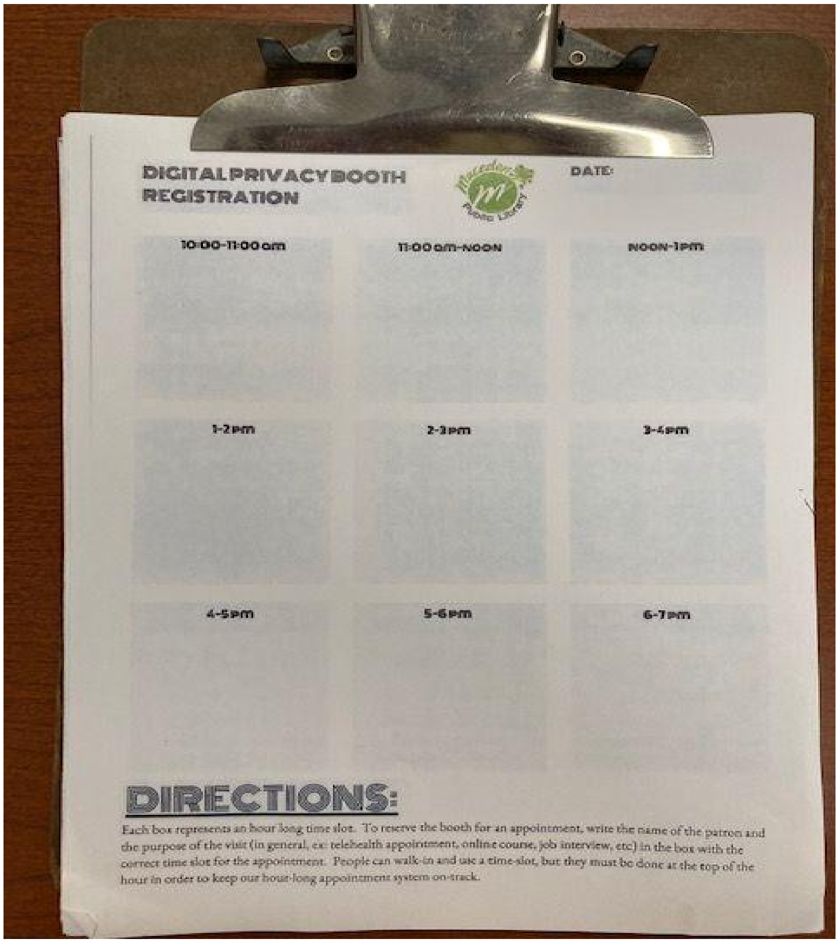

The directions state: Each box represents an hour long time slot. To reserve the booth for an appointment, write the name of the patron and the purpose of the visit (in general, ex: telehealth appointment, online course, job interview, etc.) in the box with the correct time slot for the

appointment. People may walk-in and use a time slot, but they must be done at the top of the hour in order to keep our hour-long appointment system on-track.*

Please note: *We did not receive an overwhelming number of appointments at any one time and adhering to a strict hour-long appointment did not end up being necessary.*

REVIEW OF BOOTH AS AN ASSET:
The booth, although not tremendously busy, has been an asset to the community. For anyone needing a private spot to work or meet virtually, it has definitely served its purpose well. For patrons who have made digital literacy appointments with Literacy Rochester volunteers, the booth offered a great spot to receive help.

When the library heard about this opportunity available through Rochester Regional Library Council, we jumped at the chance to have a digital privacy booth to offer access to telehealth care to patrons. Having access to medical care at the library has been the priority from inception.

Macedon is an aging community. Driving into the city of Rochester or east to Newark can be a huge obstacle to seniors who are nervous about driving in the inclement weather we receive from November through April or in the congested city traffic. It is also difficult for some to sit in a waiting room for long lengths of time waiting for an appointment. Although being able to offer quality physical medical care to senior citizens was one of the library's goals for the booth, another prime goal was to offer mental health services to people of all ages in a safe and private space. Wayne County has a scarcity of mental health providers, so offering virtual mental health appointments would be one way to help connect people to potentially life saving care.

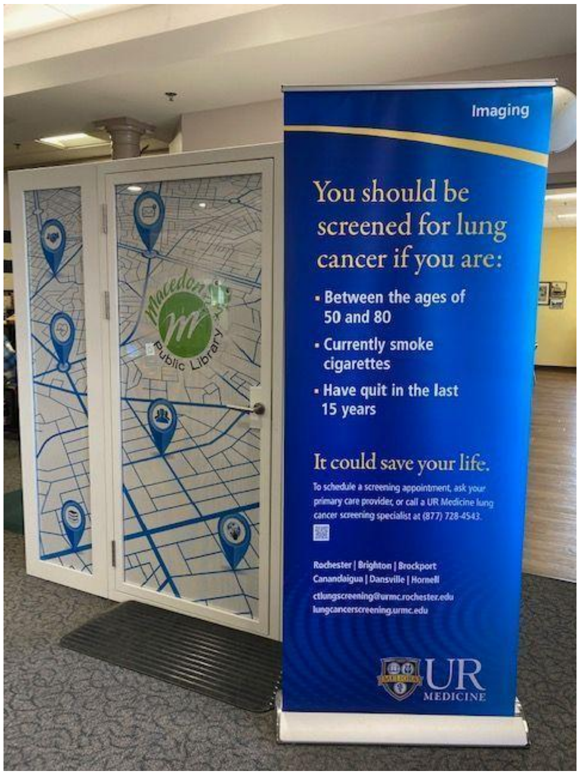

To date, the library has done the most work on creating awareness of the booth for cancer related telehealth services thanks to a partnership with the University of Rochester's Community Cancer Action Council (CCAC).

Currently, the library is working with CCAC to promote usage of the booth among people in the community who may not have reliable access to transportation into the city to receive cancer screenings, check-ins and/or consultations. We currently have a banner next to the booth to promote the availability of lung screening consultations to smokers and ex-smokers. Wayne County has a very high number of smokers and a high rate of lung cancer. We hope

to create awareness of free lung screening consultations among those who are or have smoked in order to prevent future deaths from smoking related lung cancers. We are working toward having an event this summer that creates more awareness of the booth and lung screening consultations.

Last fall before Thanksgiving, the library also highlighted the booth as a spot for telehealth appointments in a festival we called the “Thanks-Living Festival.” Local healthcare providers in a variety of fields such as: pulmonology, physical therapy, homeopathic medicine and healthcare navigation were in attendance. The festival was well received and did create more awareness of the booth for telehealth as well as other purposes.

The booth also led us to develop a partnership with Rochester Regional Healthcare. This local hospital system offers a device called a Tyto that can be used in combination with a smartphone app to take part in virtual urgent care services. The Tyto has been used by several of the library staff members inside of the booth for help with some minor health issues. We have not had patrons take advantage of this service yet, but will continue to promote it as a viable means of receiving help.

The booth has been promoted to our local representatives. Assemblyman Manktelow funded the purchase of a chair, desk, “All-in-One” desktop computer with a high definition camera and headset for listening privately. There is an unfortunate aspect of targeting the booth for telehealth appointments. During the pandemic, telehealth use became quite widespread thanks to the emergency declarations in place at the federal and state levels. Now that these declarations have expired, there are tremendous billing obstacles that preclude people from viably using telehealth options. I have spoken with both Congresswoman Tenney and Senator Gillibrand about the digital privacy booth and advocated for the removal of billing obstacles that prevent members of the community most in need of telehealth services from receiving these services. Both agreed telehealth billing needs to be remediated so telehealth services can be expanded to people who encounter barriers to quality healthcare.

USAGE:

As noted earlier, the use of the booth for technology skills training has been the most common type of appointment. Typically, senior citizens book appointments with Rochester Literacy volunteers to learn how to use their cellphone, search the internet, create an email account or to learn any sort of technical skill. The seniors are very appreciative to have a place and space to receive this help.

Other uses tend to be focused on virtual meetings, webinars and academic classes. In these cases, it is generally patrons midway between the fourth and fifth decades of life who request an appointment. Some have commented on how pleasant it is to have a space that is quiet, but not claustrophobic thanks to the customized screen that prevents them from being visible to those outside the booth, but allows them to still be able to see out while inside.

FUTURE PLANS:

We plan on continuing to promote the booth to patrons for all uses requiring a quiet, private space. We will continue our valuable collaboration with CCAC and work on offering future events that highlight the booth for telehealth purposes. We will also continue to advocate for legislation that eliminates billing obstacles to quality healthcare for all people. We know the booth can be an invaluable resource for the eldest members of our community. We also continue to look for opportunities to target the booth for mental health services.
